# High levels of TDO2 in relation to pro-inflammatory cytokines in synovium and synovial fluid of patients with osteoarthritis

**DOI:** 10.1186/s12891-022-05567-4

**Published:** 2022-06-22

**Authors:** Genxiang Rong, Tao Zhang, Yayun Xu, Zhenyu Zhang, Binjie Gui, Kongzu Hu, Jinling Zhang, Zhi Tang, Cailiang Shen

**Affiliations:** 1 Department of Orthopedics, The First Affiliated Hospital of Anhui Medical University, 218 Jixi Road, Anhui 230022 Hefei, China; 2 The Key Laboratory of Major Autoimmune Diseases of Anhui Province, Anhui Institute of Innovative Drugs, School of Pharmacy, Anhui Medical University, Hefei, China; 3 The Key Laboratory of Anti-Inflammatory and Immune Medicines, Ministry of Education, Hefei, China

**Keywords:** Osteoarthritis, Tryptophan 2,3-dioxygenase, Synovial fluid, Synovium, Pro-inflammatory cytokines

## Abstract

**Background:**

Tryptophan 2,3-dioxygenase (TDO2) is the primary enzyme that catabolizes tryptophan to kynurenine. Numerous studies have suggested that TDO2 is involved in inflammation-related diseases. However, its role in osteoarthritis (OA) has not yet been investigated. The aim of the present study was to explore the levels of TDO2 in the synovium and synovial fluid (SF) of patients with OA and its correlation with clinical manifestations and levels of pro-inflammatory cytokines.

**Methods:**

Synovium and SF samples were collected from patients with OA and patients with joint trauma (controls) during surgery. An enzyme-linked immunosorbent assay (ELISA) was used to measure TDO2, interleukin-1β (IL-1β), and tumor necrosis factor-α (TNF-α) levels in the synovium and SF. Diagnostic performance of TDO2 in the synovium to discriminate between controls and OA patients was assessed using receiver operating characteristic (ROC) curve analysis. Correlations between TDO2 levels, OA clinical features, and pro-inflammatory cytokines were evaluated using Pearson correlation analysis. Effects of IL-1β or TNF-α stimulation on TDO2 expression in OA-fibroblast-like synoviocytes (OA-FLS) were also examined.

**Results:**

The levels of TDO2, IL-1β, and TNF-α in the synovium of patients with OA were found to be significantly higher than those in controls. ROC curve analysis revealed an area under the curve (AUC) of 0.800 with 64.3% sensitivity and 85.0% specificity of TOD2 in the synovium, which enabled discriminating patients with OA from controls. Moreover, protein expression of TDO2 was upregulated to a greater extent in OA-FLS than in normal synovial fibroblasts (NSF). Furthermore, the levels of TDO2 showed significantly positive correlation with IL-1β and TNF-α levels in the synovium and SF. TDO2 levels in the synovium were also positively correlated with the Kellgren-Lawrence score. Additionally, TDO2 protein expression was significantly increased in IL-1β‒ or TNF-α‒stimulated OA-FLS than in control FLS.

**Conclusion:**

These data indicate that highTDO2 levels in the synovium can be correlated with pro-inflammatory cytokines and severity of OA.

**Supplementary Information:**

The online version contains supplementary material available at 10.1186/s12891-022-05567-4.

## Introduction

Osteoarthritis (OA), one of the most common joint diseases worldwide, is a painful disease characterized by progressive degeneration of articular cartilage and subchondral bone alteration, accompanied by synovial inflammation [1]. Epidemiological studies have found that the cumulative incidence of symptomatic knee OA over four years among Chinese adults aged ≥ 45 years was 8.5% in a National Population Survey with a 4-year follow-up [2]. As a common cause of permanent disability, OA leads to a huge and continuously growing burden on individuals and society, as well as a reduction in the quality of life of patients [3]. Although recent in vitro and in vivo evidence suggests that age, gender, obesity, genetic susceptibility, and mechanical factors are well-established risk factors for OA [4], the fundamental mechanisms responsible for development and progression of OA have not yet been fully elucidated.

Tryptophan 2,3-dioxygenase (TDO2), encoded by the gene *TDO2*, is one of the primary enzymes that catabolizes tryptophan to kynurenine [5]. Extensive evidence suggests that inflammation is associated with induction of the kynurenine pathway [6, 7]. TDO2 in rodents has been reported to be increased following treatment with pro-inflammatory mediators such as polyinosine-polycytidylic acid and stress hormones [8, 9], suggesting that TDO2 may be involved in inflammation-related diseases. Considering that synovial inflammation is not only present in a majority of OA patients but is also actively involved in progression of the disease [10], the levels of TDO2 in the synovium and synovial fluid (SF) of OA patients, their correlation with clinical manifestations, and the levels of pro-inflammatory cytokines were investigated in the present study.

It has been well demonstrated that there is a complex interplay among cells [T cells, B cells, plasma cells, mast cells, stromal cells, fibroblast-like synoviocytes (FLS), and macrophages], and soluble immune mediators are the major players in joint inflammation [11]. FLS, the main constituent cells in the synovium, maintain homeostasis in synovial tissue extracellular matrix and SF. In OA synovitis, FLS proliferate rapidly and secrete inflammatory mediators, resulting in accelerated progression of OA. Activation of FLS by pro-inflammatory cytokines induces increased expression of inflammatory cytokines, chemokines, and matrix-degrading matrix metallopeptidases (MMPs), resulting in the destruction of articular cartilage and bone [12]. The pro-inflammatory cytokines interleukin-1β (IL-1β) and tumor necrosis factor-α (TNF-α) are well defined as critical mediators in the inflammatory process of OA [13]. TNF-α is considered to be the master element in inflammatory manifestations of synovitis, whereas IL-1β is proposed to be a crucial mediator that is responsible for destruction of joints and propagation of joint inflammation [14, 15]. Therefore, TNF-α‒ or IL-1β‒stimulated FLS model is considered a suitable cell model to examine the pathogenesis of OA in vitro in numerous studies. In the present study, TDO2 expression in IL-1β‒stimulated FLS was assessed to explore the causal relationship between TDO2 and pro-inflammatory factors.

Considering the potential relationship between TDO2 and inflammatory manifestations along with the key role of inflammation in the pathogenesis of OA, we hypothesized that the levels of TDO2 in the synovium and SF of patients with OA are likely to be altered and might also be associated with the levels of pro-inflammatory cytokines. To test this hypothesis, synovium samples were obtained from patients with OA and patients with joint trauma who underwent joint surgery. TDO2, IL-1β, and TNF-α levels in the synovium and SF were measured. Diagnostic performance of TDO2 in the synovium that helps to discriminate between controls and OA patients was assessed using receiver operating characteristic (ROC) curve analysis. Correlation between TDO2 levels, OA clinical features, and pro-inflammatory cytokines was evaluated using Pearson correlation analysis. The effect of IL-1β and TNF-α stimulation on TDO2 expression in OA-FLS was also studied.

## Materials and methods

### Patients and samples

Synovium and SF samples were obtained from patients with knee OA (n = 41) who had undergone joint surgery at the First Affiliated Hospital of Anhui Medical University. Patients with OA were diagnosed according to criteria laid down by the American College of Rheumatology [16]. Patients with knee joint trauma (meniscal injury) who underwent surgery within one week were included in the control group (*n* = 20); the synovium was collected during the joint surgery. Patients with autoimmune diseases, malignancies, or infectious diseases were excluded from the control group.

### Isolation and culture of synovial fibroblasts

Synovial tissues were collected from three patients with OA who underwent knee replacement surgery (OA-FLS), and nonarthritic synovial tissues were obtained from three healthy donors who underwent arthroscopy after knee joint trauma (normal synovial fibroblasts, NSF). Knee synovial tissues were minced into small pieces of approximately 1 mm^3^ under sterile conditions, as described previously [17, 18]. The tissue was adsorbed using a pipette and evenly attached to the walls of a cell culture flask. The cell culture flask was placed vertically in a cell incubator at 37 °C and 5% CO_2_, and then placed horizontally after 6 h. The culture medium was composed of 80% DMEM/F12 (Hyclone, USA) and 20% fetal bovine serum (FBS, Gibco, USA), supplemented with 100 IU/mL penicillin and 100 μg/mL streptomycin. The FLS were passaged 3 to 5 times before use. Recombinant human cytokines IL-1β and TNF-α were obtained from eBioscience Inc (San Diego, CA, USA). OA-FLS were stimulated with IL-1β (10 ng/ml) or TNF-α (10 ng/ml) for 24 h. Next, the cells were collected for western blot analysis.

### Demographic data and sample collection

Demographic information of participants was collected using a self-designed demographic questionnaire that was designed for the present study, including age, BMI, sex, Kellgren-Lawrence score, time of pain, and time of worsening of pain. After surgery, the synovium and SF samples were immediately frozen in liquid nitrogen and transferred to a -80 °C freezer for storage until use.

### ELISA analysis

For tissue ELISA, synovium samples (100 mg) were homogenized in 1 ml of complete Mini protease-inhibitor cocktail homogenization buffer (Roche, Indianapolis, IN) on ice, followed by sonication for 30 s. The homogenates were centrifuged and filtered through a 0.45 μm pore size filter, and the levels of TDO2, IL-1β, and TNF-α were quantified by ELISA. The final protein concentrations of TDO2, IL-1β, and TNF-α in the synovium were normalized to the protein concentration in each tissue using the DC protein assay (Bio-Rad Laboratories, Hercules, CA), according to the manufacturer’s protocol. Furthermore, the TDO2, IL-1β, and TNF-α levels in the synovium and SF were measured using commercially available ELISA kits (Jianglai Bio, Shanghai, China), following the manufacturers' instructions. The catalog numbers of the corresponding kits are as follows: TDO2 (JL13234), IL-1β (JL13662), and TNF-α (JL10208). For TDO2 ELISA kit, the sensitivity was < 0.1 ng/ml and range of detection was 0.25‒8 ng/ml; for both the IL-1β and TNF-α ELISA kits, the sensitivity was < 0.1 pg/ml and range of detection was 2.5‒80 pg/ml.

### Immunofluorescence staining

Synovium samples from five patients with OA and five patients with knee joint trauma were harvested and immediately fixed in 4% paraformaldehyde for 48 h, embedded in paraffin, and cut into 4 µm-thick serial sections. Next, the sections underwent dewaxing, rehydration, antigen retrieval, and blocking. Subsequently, the sections were stained using TDO2 antibody (1: 400; Proteintech, USA) overnight at 4 °C. After washing with PBS (three times for 5 min), the sections were incubated with the corresponding fluorescent-labeled secondary antibody (Bioss, Beijing, China) for 1 h at 4 °C in the dark and then counterstained with 4',6-diamidino-2-phenylindole (DAPI; Beyotime Biotechnology, Nanjing, China) for 5 min. Images were captured using a fluorescence inversion microscope (Olympus, Japan). Isotype control immunofluorescence with isotype-matched IgG did not show any staining (Supplementary Fig. 1A).

### Immunohistochemical staining

Synovium samples from five patients with OA and five patients with knee joint trauma were harvested and immediately fixed in 4% paraformaldehyde for 48 h, embedded in paraffin, and cut into 4 µm-thick serial sections. Immunohistochemical staining was performed using SP-9000 Histostain-Plus kits (Zsgb Bio, China) according to the manufacturers’ protocol. Briefly, the sections were deparaffinized in xylene, rehydrated in a graded series of alcohol, and washed twice in PBS for 5 min. Endogenous peroxidase activity was quenched using 3% H_2_O_2_ for 10 min followed by antigen retrieval using 0.1% trypsin for 15 min. The sections were then blocked with normal goat serum for 30 min and incubated with TDO2 antibody (1: 400; Proteintech, USA) overnight at 4 °C. On the second day, the sections were rinsed with PBS and incubated with horseradish peroxidase-conjugated secondary antibodies (Zsgb Bio, China) for 30 min at 37 °C. Finally, the sections were stained using a diaminobenzidine (DBA) kit (Zsgb Bio, China). A digital pathology slide scanner (3DHISTECH, Digital Pathology Company, Budapest, Hungary) was used to scan the stained sections. The immunohistochemical staining results were quantitatively analyzed using Image-Pro Plus software (Media Cybernetics, Silver Spring, MD, USA) to calculate the integral optical density (IOD). Isotype control immunohistochemistry with isotype-matched IgG showed no staining (Supplementary Fig. 1B).

### Western blot analysis

Western blot analysis was carried out to evaluate protein levels. Briefly, cells were washed twice with ice-cold PBS and lysed in RIPA buffer containing protease inhibitors and protein phosphatase inhibitors. Protein concentration was measured using a BCA Protein Assay Kit (Beyotime Biotechnology, Nanjing, China), and equal amounts of protein samples from each group were separated by 10% SDS–polyacrylamide gel electrophoresis (SDS-PAGE) and transferred onto PVDF membranes (Millipore Corp). Next, the membranes were blocked with 5% non-fat milk in Tris-buffered saline containing 0.1% tween-20 (TBST) at room temperature for 2 h, and incubated with specific primary antibodies against TDO2 (1: 1000; Proteintech, USA) and β-actin (1: 1000; Bioworld Technology Co. Ltd., Nanjing, China) overnight at 4 °C. The membranes were then washed three times with TBST and incubated with HRP-conjugated secondary antibodies (1: 10,000) for 1 h. The blots were cut prior to hybridisation with antibodies during blotting. Protein bands were detected using the ECL chemiluminescent kit (Thermo Fisher Scientific) and analyzed using ImageJ software.

### Source of microarray data

The gene expression profile dataset (GSE55235) was downloaded from the Gene Expression Omnibus (GEO) database (https://www.ncbi.nlm.nih.gov/geo). The GSE55235 dataset included 10 healthy synovium (post-mortem joint samples) and 10 OA synovium samples. Platform and series matrix file (s) were downloaded from the GEO database and saved as TXT files. The probes were transformed into the corresponding gene symbols according to the relevant annotation information provided on the platform. For gene symbols with multiple probes, the average value was used as the final expression value. The average expression of *TDO2* mRNA in synovial membrane samples from patients with OA and healthy controls was compared.

### Statistical analysis

All data were expressed as mean ± standard error of the mean (SEM) and analyzed using SPSS software (version 17.0; IBM Corp, Armonk, NY, USA), with a value of *P* < 0.05 considered to be statistically significant. Distribution of continuous variables was tested for normality using the one-sample Kolmogorov–Smirnov test. Student's t-test was used to test for differences in continuous variables, including those related to age and body mass index (BMI), between groups. To analyze sex difference between groups, the χ^2^ test was used. Analysis of covariance (ANCOVA) was performed to compare differences in TDO2, IL-1β, and TNF-α levels in the synovium between two groups, controlling for age and BMI by using these variables as covariates. To test correlation between variables, the Pearson correlation test was used.

## Results

### Comparison of demographic data, TDO2, IL-1β, and TNF-α levels in synovium and SF of OA and control groups

Table 1 shows that no significant differences in sex were found between the OA and control groups (*χ*^*2*^ = 3.43, *P* = 0.064). However, the age (*t* = -11.22, *P* < 0.001) and BMI (*t* = -2.04, *P* = 0.046) of OA patients were significantly higher than those of controls.

**Table 1:** Intergroup comparison of demographic data (mean ± SEM)

Variables	Control group (*n* = 20)	OA group (*n* = 41)	Statistics (*t*/*χ*^*2*^)	*P*
Age	42.30 ± 2.29	66.71 ± 1.12	-11.22	< 0.001
BMI (kg/m^2^)	24.80 ± 0.58	26.46 ± 0.49	-2.04	0.046
Gender (female/male)	11/9	32/9	3.43	0.064

*BMI* body mass index, *OA* Osteoarthritis, *SEM* Standard error of mean

The Kellgren-Lawrence score of OA patients was an average of 3.15 ± 0.14, with a reported time of pain ranging from 0.17 to 30 years (mean 7.89 ± 1.10 years). Serum C-reactive protein (CRP) levels and erythrocyte sedimentation rate (ESR) of OA patients were 4.96 ± 1.53 mg/L and 16.83 ± 2.11 mm/h, respectively.

### TDO2 levels in synovium of OA and control groups

**Fig. 1:**
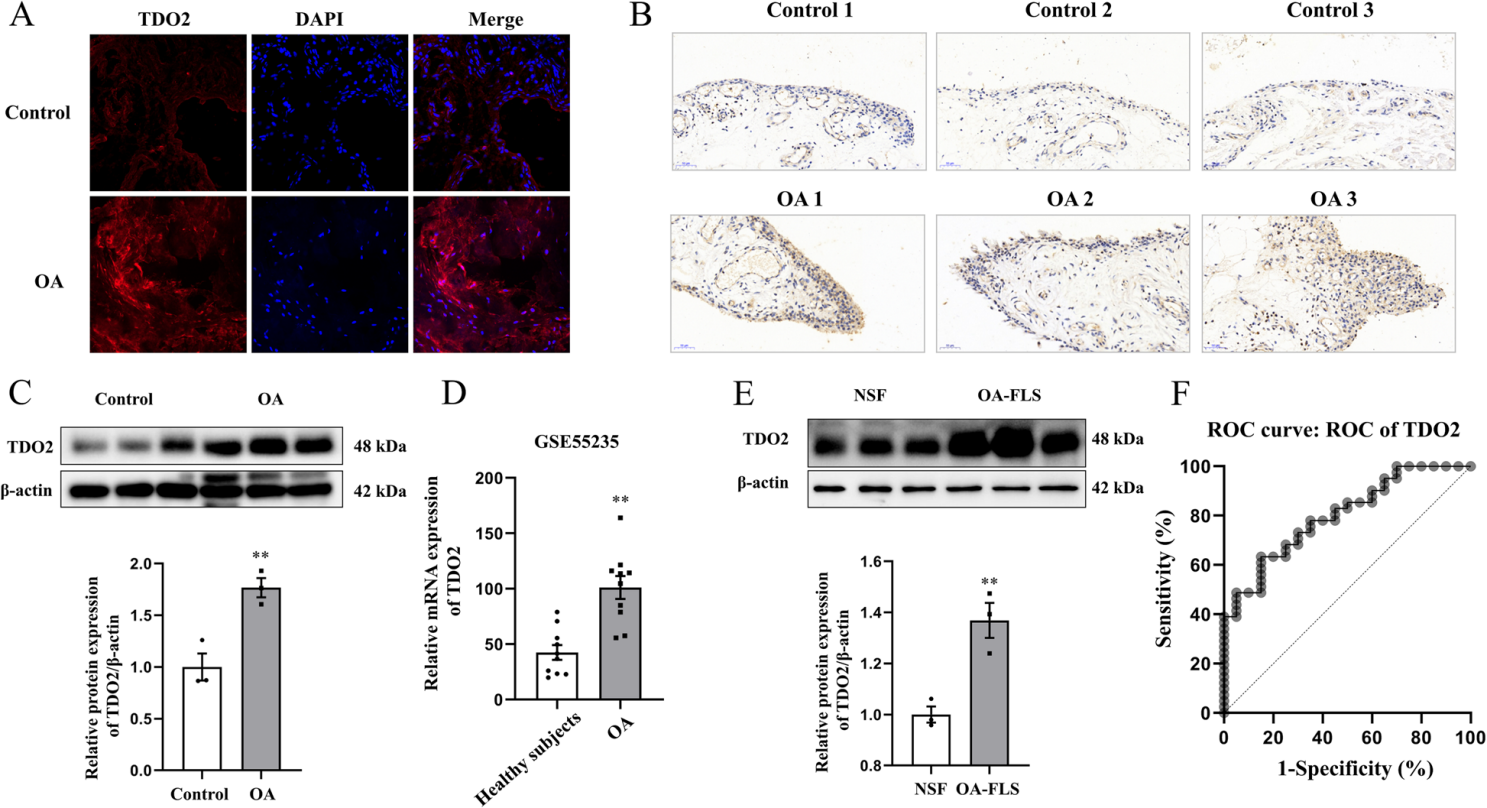
TDO2 levels in synovium of OA and control groups. (A) Typical immunofluorescence images of TDO2 protein expression in synovium of the OA group (*n* = 5) and control group (*n* = 5) (magnification, 20 ×); (B) typical immunohistochemical images of TDO2 protein expression in synovium of OA (*n* = 5) and control (*n* = 5) groups (magnification, 40 ×); (C) typical western blot images and quantitative analysis of TDO2 protein expression in synovium of the OA (*n* = 3) and control (*n* = 3) groups; (D) expression level of TDO2 mRNA in synovium of OA patients (*n* = 10) and healthy subjects (*n* = 10) (Database source: GSE 55,235); (E) typical western blot images and quantitative analysis of TDO2 protein expression in NSF (*n* = 3) and OA-FLS (*n* = 3); (F) ROC curve of TDO2 in synovium was used to identify patients with OA. Data are presented as mean ± SEM. **P < 0.01 vs. control group, healthy subjects, or NSF group

As shown in Table 2, results of the ANCOVA showed that TDO2 levels in the synovium of OA patients (*n* = 41) were significantly higher than those of controls (*n* = 20), taking into consideration age, sex, and BMI as covariates (*F* = 4.487, *P* = 0.039). The results of immunofluorescence (Fig. 1A), immunohistochemistry (Fig. 1B), and western blotting (*t* = -4.78, *P* = 0.009; Fig. 1C; uncropped images are provided in Supplementary Fig. 1C-D) showed that protein expression of TDO2 in the synovium was upregulated to a greater extent in the OA group than in control group. Moreover, TDO2 levels in the synovium were significantly higher in patients with OA than in healthy subjects, according to the NCBI GEO database (accession number: GSE55235; *t* = -4.773, *P* < 0.001; Fig. 1D). Furthermore, the protein expression of TDO2 was upregulated in OA-FLS of NSF (*t* = -4.88, *P* = 0.008; Fig. 1E; uncropped images are provided in Supplementary Fig. 1E-F). Additionally, ROC curve analysis showed that TDO2 levels in the synovium had a good potential diagnostic value in OA (Fig. 1F); the AUC for TDO2 levels was 0.800 (95% CI: 0.689‒0.911). At a cutoff point of 47.12 ng/g for TDO2, which was applied to differentiate patients with OA from controls, the sensitivity and specificity were 63.4% and 85.0%, respectively.

**Table 2:** Intergroup comparison of TDO2, IL-1β, and TNF-α levels in synovium (mean ± SEM)

Variables	Control group (*n* = 20)	OA group (*n* = 41)	Statistics (*F*)	*P*
TDO2 in synovium (ng/g)	39.20 ± 1.90	52.02 ± 1.97	4.487	0.039
IL-1β in synovium (pg/g)	419.16 ± 20.95	586.44 ± 23.15	9.17	0.004
TNF-α in synovium (pg/g)	449.17 ± 28.64	701.02 ± 18.62	21.59	< 0.001

*IL-1β* interleukin-1β, *SEM* Standard error of mean, *TDO2* Tryptophan 2,3-dioxygenase, *TNF-α* Tumor necrosis factor-α, *OA* Osteoarthritis

Sex and BMI were used as covariates

### Pro-inflammatory cytokines (IL-1β and TNF-α) levels in synovium of OA and control groups

Regarding pro-inflammatory cytokines, IL-1β (*F* = 9.17, *P* = 0.004) and TNF-α (*F* = 21.59, *P* < 0.001) levels in the synovium were significantly higher in the OA group than in control group, using age, sex, and BMI as covariates (Table 2).

### Relationship between TDO2 levels in synovium and SF and clinical features in the OA group

**Fig. 2:**
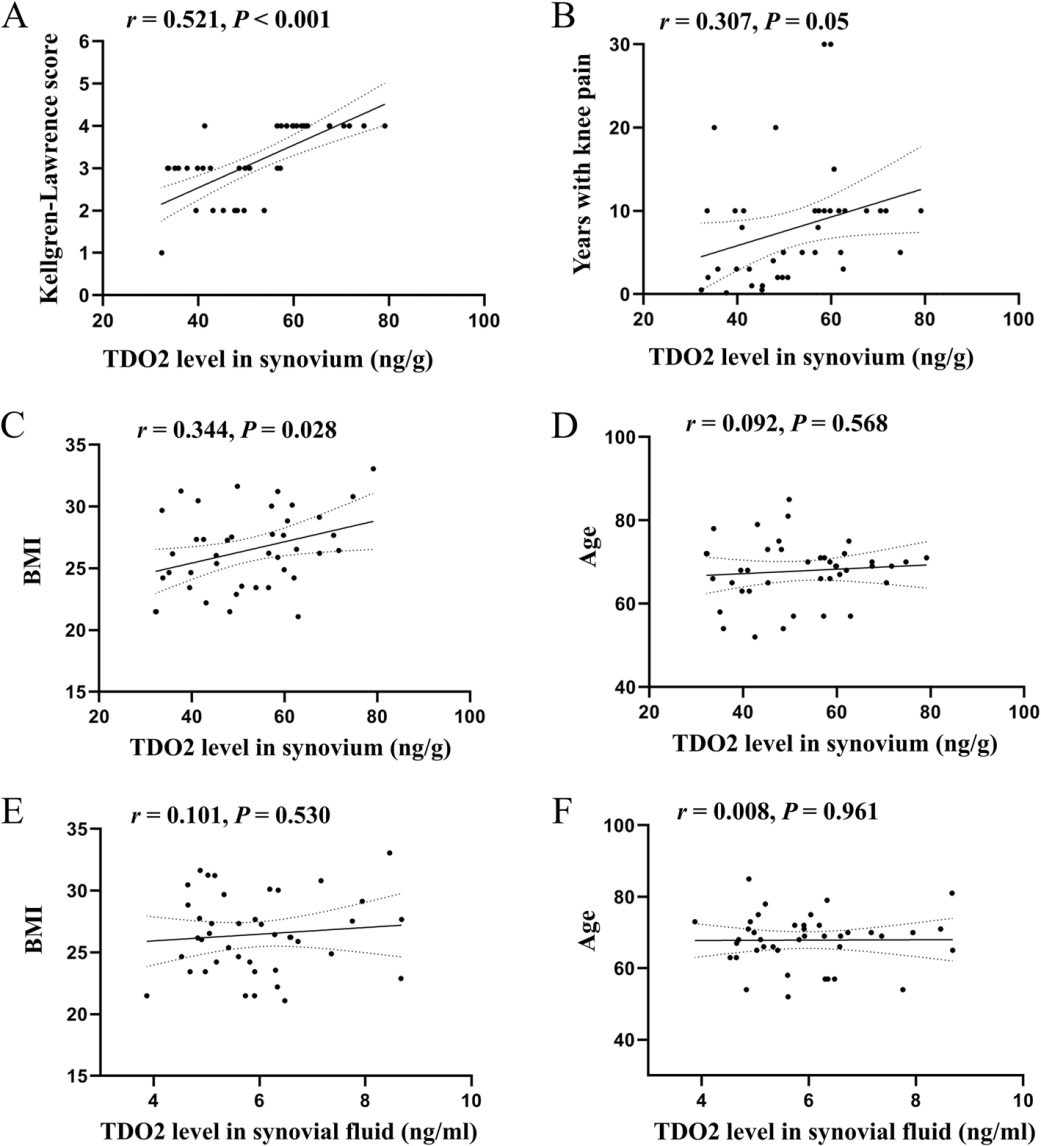
Correlation between TDO2 levels in synovium and SF and clinical features in the OA group. Correlation between TDO2 levels in synovium and Kellgren-Lawrence score (A), time duration (years) in patients with knee pain (B), BMI (C), and age (D); correlation between TDO2 levels in SF and (E) BMI and (F) age. *P* < 0 .05 was considered to be statistically significant

As shown in Fig. 2A, TDO2 levels in the synovium were positively correlated with the Kellgren-Lawrence score in patients with OA (*r* = 0.521, *P* < 0.001). Moreover, a positive relationship was found between TDO2 levels in the synovium and time of pain measured in years in OA patients (*r* = 0.307, *P* = 0.05; Fig. 2B). Furthermore, TDO2 levels in the synovium were positively correlated with BMI (*r* = 0.344, *P* = 0.028; Fig. 2C), but not with age (*r* = 0.092, *P* = 0.568; Fig. 2D) in OA patients. However, no correlation between TDO2 levels in the SF and BMI (*r* = 0.101, *P* = 0.530; Fig. 2E) or age (*r* = 0.008, *P* = 0.961; Fig. 2F) was observed in patients with OA.

### Relationship between TDO2 levels in synovium and SF and pro-inflammatory cytokines in the OA group

**Fig. 3:**
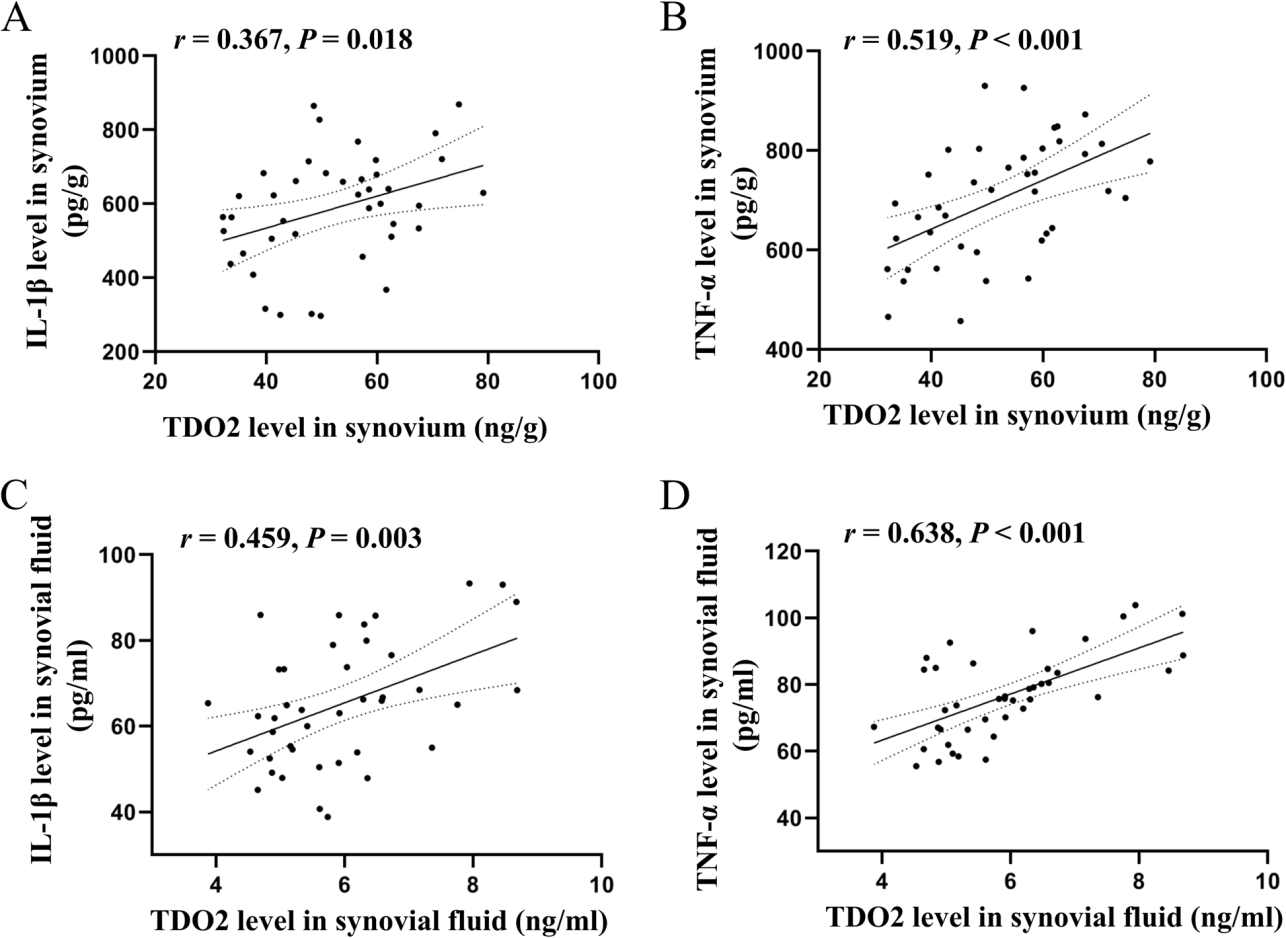
Correlation between TDO2 levels in synovium and SF and pro-inflammatory cytokines in the OA group. Correlation between TDO2 and IL-1β levels (A) and TNF-α levels (B) in synovium; correlation between TDO2 and IL-1β levels (C) and TNF-α levels (D) in SF. *P* < 0.05 was considered to be statistically significant

Figures 3A‒B show that TDO2 levels were positively correlated with IL-1β (*r* = 0.367, *P* = 0.018) and TNF-α (*r* = 0.519, *P* < 0.001) levels in the synovium of patients with OA. Similarly, the levels of TDO2 showed a significantly positive correlation with IL-1β (*r* = 0.459, *P* = 0.003) and TNF-α levels (*r* = 0.638, *P* < 0.001) in the SF of patients with OA (Figs. 3C‒D).

### TDO2 protein expression in IL-1β or TNF-α‒stimulated OA-FLS

**Fig. 4:**
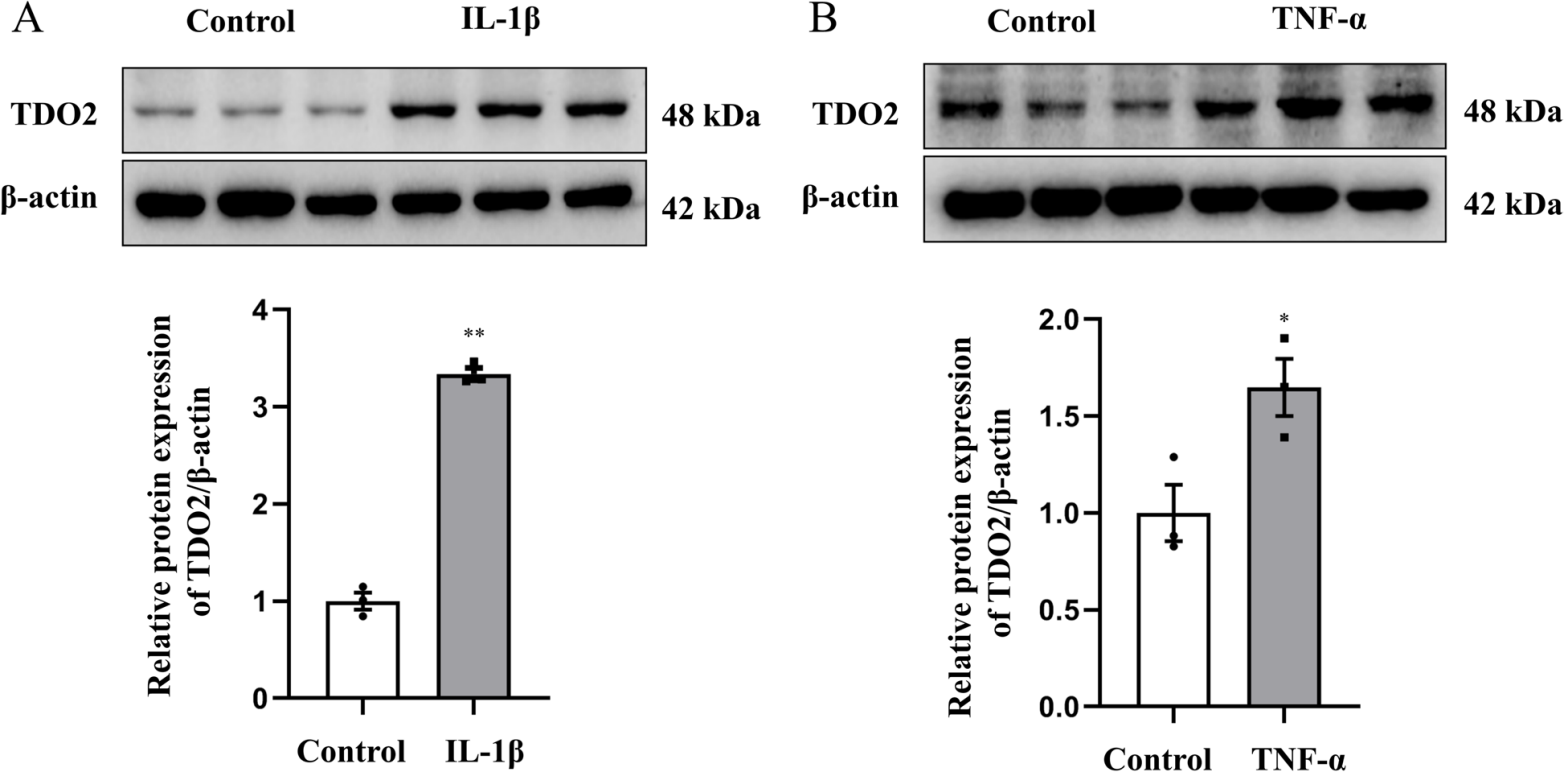
TDO2 protein expression in IL-1β‒ and TNF-α‒stimulated OA-FLS. (A) Typical western blotimages and quantitative analysis of TDO2 protein expression in IL-1β‒stimulated OA-FLS (*n* = 3) and controls (*n* = 3). (B) typical western blotimages and quantitative analysis of TDO2 protein expression in TNF-α‒stimulated OA-FLS (*n* = 3) and controls (*n* = 3); **P < 0.01, *P < 0.05 vs. control group

Figure 4 shows that the TDO2 protein expression was significantly increased in IL-1β‒ (*t* = -21.557, *P* < 0.001; Fig. 4A; uncropped images are provided in Supplementary Fig. 2A-B) or TNF-α‒stimulated (*t* = -3.127, *P* = 0.035; Fig. 4B; uncropped images are provided in Supplementary Fig. 2C-D) OA-FLS than in controls.

## Discussion

In the present study, we demonstrated that the average levels of TDO2, IL-1β, and TNF-α in the synovium were significantly higher in patients with OA than in controls. Results of the ROC curve analysis revealed an AUC of 0.800 with 64.3% sensitivity and 85.0% specificity of TDO2 in the synovium, which was used to discriminate patients with OA from controls. Moreover, protein expression of TDO2 was upregulated in OA-FLS than in NSF. Furthermore, the levels of TDO2 showed a significantly positive correlation of IL-1β and TNF-α levels between the synovium and SF. TDO2 levels in the synovium were also positively correlated with the Kellgren-Lawrence score and time of pain. Additionally, TDO2 protein expression was significantly increased in IL-1β‒ or TNF-α‒stimulated OA-FLS than in controls. Collectively, these results suggest that increased TDO2 levels in the synovium may be associated with pro-inflammatory cytokines and severity of OA.

A close link between obesity and OA has been widely demonstrated [19]. Epidemiological data show that subjects with BMI > 30 kg/m^2^ have 6.8 times higher chances of developing knee OA than those displaying normal weight [20]. A high BMI is associated with faster disease progression [21], suggesting that obesity is associated with an increased risk of functional impairment in patients with knee OA. Moreover, older age has been reported to be another major risk factor for OA [22]. The prevalence of radiographic knee OA increased from 26.2% among participants in the range of 55‒64 years to nearly 50% of those who were older than 75 years [23]. In the present study, both the age and BMI of patients with OA were significantly higher than those of controls. Moreover, TDO2 levels in the synovium were positively correlated with BMI in patients with OA. To eliminate the possible influences of age, sex, and BMI on TDO2 and pro-inflammatory cytokine levels in the synovium and SF, ANCOVA was performed to compare TDO2 and pro-inflammatory cytokine levels in the synovium between the two groups, using variables such as age, sex, and BMI as covariates.

It has been demonstrated that TDO2 expression in the brain increases with age; TDO2 is shown to be highly expressed in the brains of patients with Alzheimer's disease, an age-related neurodegenerative disorder characterized by neuronal loss and dementia [24]. This finding suggests a close relationship between age and TDO2. However, in the present study, results of the correlation analysis showed that there was no relationship between age and TDO2 levels in the SF and synovium of patients with OA. A relatively small sample size was used in our study; therefore, multicentric and longitudinal studies are required to validate the potential role of age on TDO2 levels in SF and synovial tissue of OA patients.

TDO2 is a heme-containing dioxygenase enzyme that catalyzes the first and rate-limiting step of the kynurenine pathway, namely, the conversion of tryptophan to formyl-kynurenine [25]. Recent studies have demonstrated that TDO2 is expressed in diverse tumor types, including hepatocellular carcinoma, non-small cell lung cancer, ovarian carcinoma, and renal cell carcinoma [26, 27]. Functionally, TDO2 promotes tumor cell motility and suppresses T cell proliferation and function [28]. More recently, TDO2 expression was reported to be strongly increased in the synovial tissue and FLS of rheumatoid arthritis patients and adjuvant-induced arthritic rats [29]. Nevertheless, till date, TDO2 levels in patients with OA have not been investigated. The present study is the first to show that the average TDO2 levels were significantly increased in the synovium of patients with OA and that the protein expression of TDO2 was upregulated in OA-FLS. Correlation analysis demonstrated that TDO2 levels in the synovium were also positively correlated with Kellgren-Lawrence score and duration in years of knee pain. These results suggest that TDO2is associated with disease severity and plays a crucial role in the pathogenesis of OA. Although the results of ROC curve analysis revealed an AUC of 0.800 for TDO2 in the synovium, which helped in discriminating patients with OA from controls, the diagnostic value of synovial TDO2 levels due to an increase in the levels of TDO2 in different joint diseases in OA patients needs to be further confirmed [29].

Accumulated data support an undeniable link between TDO2 and inflammatory response. Recent research has revealed that chronic inflammation causes an increase in inflammatory cytokine levels and results in activation of the kynurenine pathway [30, 31]. In the present study, Pearson correlation analysis consistently showed that TDO2 levels were positively correlated with IL-1β and TNF-α levels in the synovium and SF of patients with OA, thereby indicating that the increased TDO2 levels in OA patients might be associated with an increase in pro-inflammatory cytokines. Recently, IL-1β has been implicated to induce the production of proinflammatory cytokines, including IL-6 and IL-8, by increasing TDO2 expression in endometriosis [32]. Taking into account the results of the present study that IL-1β or TNF-α stimulation could increase the TDO2 protein expressions in OA-FLS, it is rational to presume that TDO2 may be involved in the occurrence and development of OA caused by inflammation.

Immunohistochemistry and immunofluorescence assays revealed that TDO2 was mainly expressed in lining layers of the synovium, where synovial fibroblasts are located [33]. However, inflammatory cells (mostly lymphocytes and plasma cells) present in the sublining layers exhibited little or no expression of TDO2. Thus, in the present study, we compared the differences in the expression of TDO2 levels in OA-FLS and NSF. The results showed that protein expression of TDO2 was upregulated to a greater extent in OA-FLS than in NSF. Nevertheless, further studies are needed to assess the expression of TDO2 in various types of cells of the synovial tissue.

It has been demonstrated that macrophages are the main component of SF cells, followed by T cells, in OA [34, 35]. More recently, immune cells, including macrophages, were found to express high amounts of TDO2 proteins at the peak stage of adjuvant-induced arthritis in a rat model of rheumatoid arthritis [29]. Therefore, we speculate that TDO2 in SF may partly originate from immune cells present in SF; nonetheless, this finding needs to be confirmed by further research.

There are some limitations to this study. First, it is a single-center study with a small sample size. Additional studies with larger sample sizes are needed to investigate the differences in TDO2 levels among different OA subtypes. Second, specific correlation between biomarkers of inflammation and knee OA remains controversial; in addition, this study only explored the relationship between TDO2 and IL-1β or TNF-α. Hence, the effect of TDO2 on OA-FLS needs to be further explored. Third, since a positive relationship between TDO2 levels in the synovium and Kellgren-Lawrence score was observed, a causal relationship between the two should be validated. Fourth, the two groups were not BMI- or age-matched. Fifth, due to the limited number of SF samples, we only measured TDO2, IL-1β, and TNF-α levels in this study. Other parameters, such as measurement of kynurenine levels, an indicator of TDO2 activity, should be evaluated.

## Conclusion

We conclude that increased TDO2 levels in the synovium may be associated with pro-inflammatory cytokines and severity of OA. However, multicentric and longitudinal studies are required to validate the potential role of TDO2 in the pathogenesis of OA and the possibility of using TDO2 as a potential target for OA treatment.

## Supplementary Information

**Additional file 1:** **SupplementaryFigure 1. **Isotype controls for immunofluorescence staining andimmunohistochemistry staining and raw data of western blot inFigure 1.(A) representativephoto of the isotype control for immunofluorescence staining;(B) representativephoto of the isotype control for immunohistochemistry staining; (C) the original gels showing TDO2 expression in Fig 1C; (D) the original gels showing β-actinexpression in Fig 1C. (E) theoriginal gels showing TDO2 expression in Fig 1E; (F) the original gels showing β-actinexpression in Fig 1E. Since our original exposure was very bright, there was no way to changeit, and we could not see the edges of the gels by adjusting the contrast in theoriginal gels of western blot.

**Additional file 2:** **SupplementaryFigure 2. **Raw data of western blot in Figure 4. (A) the original gels showing TDO2 expression inFig 4A; (B) the original gelsshowing β-actin expression in Fig 4A; (C) the original gels showing TDO2 expression in Fig 4B; (D) the original gels showing β-actinexpression in Fig 4B.

## Data Availability

The data used to support the findings of this study will be available with the request to the corresponding author.

## Abbreviations

TDO2: Tryptophan 2,3-dioxygenase
OA: Osteoarthritis
SF: Synovial fluid
ELISA: Enzyme-linked immunosorbent assay
IL-1β: Interleukin-1β
TNF-α: Tumor necrosis factor-α
ROC: Receiver operating characteristic
FLS: Fibroblast-like synoviocytes
AUC: Area under curve
NSF: Normal synovial fibroblasts
MMPs: Matrix-degrading matrix metallopeptidases
PAGE: Polyacrylamide gel electrophoresis
SEM: Standard error of the mean
BMI: Body mass index
ANCOVA: Analysis of covariance
CRP: C-reactive protein

## References

[1] Hu Y, Chen X, Wang S, Jing Y, Su J (2021). Subchondral bone microenvironment in osteoarthritis and pain. Bone research.

[2] Ren Y, Hu J, Tan J, Tang X, Li Q, Yang H, Liu C, He Q, Zou K, Sun X (2020). Incidence and risk factors of symptomatic knee osteoarthritis among the Chinese population: analysis from a nationwide longitudinal study. BMC Public Health.

[3] Vaughn I, Terry E, Bartley E, Schaefer N, Fillingim R (2019). Racial-Ethnic Differences in Osteoarthritis Pain and Disability: A Meta-Analysis. J Pain.

[4] O'Neill T, McCabe P, McBeth J (2018). Update on the epidemiology, risk factors and disease outcomes of osteoarthritis. Best Pract Res Clin Rheumatol.

[5] Badawy A (2017). Kynurenine Pathway of Tryptophan Metabolism: Regulatory and Functional Aspects. International journal of tryptophan research : IJTR.

[6] Song P, Ramprasath T, Wang H, Zou M (2017). Abnormal kynurenine pathway of tryptophan catabolism in cardiovascular diseases. Cellular and molecular life sciences : CMLS.

[7] Joisten N, Kummerhoff F, Koliamitra C, Schenk A, Walzik D, Hardt L, Knoop A, Thevis M, Kiesl D, Metcalfe A (2020). Exercise and the Kynurenine pathway: Current state of knowledge and results from a randomized cross-over study comparing acute effects of endurance and resistance training. Exerc Immunol Rev.

[8] Brooks A, Lawson M, Rytych J, Yu K, Janda T, Steelman A, McCusker R (2016). Kynurenine PathwayImmunomodulatory Factors Galectin-9 and Interferon-Gamma Synergize to Induce Expression of Rate-Limiting Enzymes of the in the Mouse Hippocampus. Front Immunol.

[9] Brooks A, Lawson M, Smith R, Janda T, Kelley K, McCusker R (2016). Interactions between inflammatory mediators and corticosteroids regulate transcription of genes within the Kynurenine Pathway in the mouse hippocampus. J Neuroinflammation.

[10] van den Bosch M (2019). Inflammation in osteoarthritis: is it time to dampen the alarm(in) in this debilitating disease?. Clin Exp Immunol.

[11] Smolinska V, Debreova M, Culenova M, Csobonyeiova M, Svec A, Danisovic L (2022). Implication of Mesenchymal Stem Cells and Their Derivates for Osteochondral Regeneration. Int J Mol Sci.

[12] Lefèvre S, Knedla A, Tennie C, Kampmann A, Wunrau C, Dinser R, Korb A, Schnäker E, Tarner I, Robbins P (2009). Synovial fibroblasts spread rheumatoid arthritis to unaffected joints. Nat Med.

[13] Wojdasiewicz P, Poniatowski Ł, Szukiewicz D (2014). The role of inflammatory and anti-inflammatory cytokines in the pathogenesis of osteoarthritis. Mediators Inflamm.

[14] van den Berg W (2000). Arguments for interleukin 1 as a target in chronic arthritis. Ann Rheum Dis.

[15] Zwerina J, Redlich K, Polzer K, Joosten L, Krönke G, Distler J, Hess A, Pundt N, Pap T, Hoffmann O (2007). TNF-induced structural joint damage is mediated by IL-1. Proc Natl Acad Sci USA.

[16] Altman R, Alarcón G, Appelrouth D, Bloch D, Borenstein D, Brandt K, Brown C, Cooke T, Daniel W, Feldman D (1991). The American College of Rheumatology criteria for the classification and reporting of osteoarthritis of the hip. Arthritis Rheum.

[17] Bartok B, Firestein G (2010). Fibroblast-like synoviocytes: key effector cells in rheumatoid arthritis. Immunol Rev.

[18] Zhang Y, Qian X, Yang X, Niu R, Song S, Zhu F, Zhu C, Peng X, Chen F (2020). ASIC1a induces synovial inflammation via the Ca/NFATc3/ RANTES pathway. Theranostics.

[19] Messier S (2009). Obesity and osteoarthritis: disease genesis and nonpharmacologic weight management. Med Clin North Am.

[20] Coggon D, Reading I, Croft P, McLaren M, Barrett D, Cooper C (2001). Knee osteoarthritis and obesity. IJO.

[21] Raynauld J, Martel-Pelletier J, Berthiaume M, Beaudoin G, Choquette D, Haraoui B, Tannenbaum H, Meyer J, Beary J, Cline G (2006). Long term evaluation of disease progression through the quantitative magnetic resonance imaging of symptomatic knee osteoarthritis patients: correlation with clinical symptoms and radiographic changes. Arthritis Res Ther.

[22] Shane Anderson A, Loeser R (2010). Why is osteoarthritis an age-related disease?. Best Pract Res Clin Rheumatol.

[23] Jordan J, Helmick C, Renner J, Luta G, Dragomir A, Woodard J, Fang F, Schwartz T, Abbate L, Callahan L (2007). Prevalence of knee symptoms and radiographic and symptomatic knee osteoarthritis in African Americans and Caucasians: the Johnston County Osteoarthritis Project. J Rheumatol.

[24] Wu W, Nicolazzo J, Wen L, Chung R, Stankovic R, Bao S, Lim C, Brew B, Cullen K, Guillemin G (2013). Expression of tryptophan 2,3-dioxygenase and production of kynurenine pathway metabolites in triple transgenic mice and human Alzheimer's disease brain. PLoS ONE.

[25] Platten M, Nollen E, Röhrig U, Fallarino F, Opitz C (2019). Tryptophan metabolism as a common therapeutic target in cancer, neurodegeneration and beyond. Nat Rev Drug Discovery.

[26] Opitz C, Litzenburger U, Sahm F, Ott M, Tritschler I, Trump S, Schumacher T, Jestaedt L, Schrenk D, Weller M (2011). An endogenous tumour-promoting ligand of the human aryl hydrocarbon receptor. Nature.

[27] Pilotte L, Larrieu P, Stroobant V, Colau D, Dolusic E, Frédérick R, De Plaen E, Uyttenhove C, Wouters J, Masereel B (2012). Reversal of tumoral immune resistance by inhibition of tryptophan 2,3-dioxygenase. Proc Natl Acad Sci USA.

[28] Mohapatra S, Sadik A, Tykocinski L, Dietze J, Poschet G, Heiland I, Opitz C (2019). Hypoxia Inducible Factor 1α Inhibits the Expression of Immunosuppressive Tryptophan-2,3-Dioxygenase in Glioblastoma. Front Immunol.

[29] Chang Y, Han P, Wang Y, Jia C, Zhang B, Zhao Y, Li S, Li S, Wang X, Yang X et al. Tryptophan 2,3-dioxygenase 2 plays a key role in regulating the activation of fibroblast-like synoviocytes in autoimmune arthritis. Br J Pharmacol. 2021;179(12):3024–42. 10.1111/bph.15787 34969166

[30] Quak J, Doornbos B, Roest A, Duivis H, Vogelzangs N, Nolen W, Penninx B, Kema I, de Jonge P (2014). Does tryptophan degradation along the kynurenine pathway mediate the association between pro-inflammatory immune activity and depressive symptoms?. Psychoneuroendocrinology.

[31] Myint A, Kim Y, Verkerk R, Scharpé S, Steinbusch H, Leonard B (2007). Kynurenine pathway in major depression: evidence of impaired neuroprotection. J Affect Disord.

[32] Urata Y, Koga K, Hirota Y, Akiyama I, Izumi G, Takamura M, Nagai M, Harada M, Hirata T, Yoshino O (2014). IL-1β increases expression of tryptophan 2,3-dioxygenase and stimulates tryptophan catabolism in endometrioma stromal cells. Am J Reprod Immunol.

[33] Masoumi M, Bashiri H, Khorramdelazad H, Barzaman K, Hashemi N, Sereshki H, Sahebkar A, Karami J (2021). Destructive Roles of Fibroblast-like Synoviocytes in Chronic Inflammation and Joint Damage in Rheumatoid Arthritis. Inflammation.

[34] Gómez-Aristizábal A, Gandhi R, Mahomed N, Marshall K, Viswanathan S (2019). Synovial fluid monocyte/macrophage subsets and their correlation to patient-reported outcomes in osteoarthritic patients: a cohort study. Arthritis Res Ther.

[35] Cunha J, Barbosa G, Castro P, Luiz B, Silva A, Russo T, Vasilceac F, Cunha T, Cunha F, Salvini T (2019). Knee osteoarthritis induces atrophy and neuromuscular junction remodeling in the quadriceps and tibialis anterior muscles of rats. Sci Rep.

