# Supplementary material for: High levels of TDO2 in relation to pro-inflammatory cytokines in synovium and synovial fluid of patients with osteoarthritis
Source: BMC Musculoskelet Disord. 2022 Jun 22;23:604. doi: 10.1186/s12891-022-05567-4 (PMC9214984; doi:10.1186/s12891-022-05567-4)
Supplement: Supplementary file 1 — Additional file 1: SupplementaryFigure 1. Isotype controls for immunofluorescence staining andimmunohistochemistry staining and raw data of western blot inFigure 1.(A) representativephoto of the isotype control for immunofluorescence staining;(B) representativephoto of the isotype control for immunohistochemistry staining; (C) the original gels showing TDO2 expression in Fig 1C; (D) the original gels showing β-actinexpression in Fig 1C. (E) theoriginal gels showing TDO2 expression in Fig 1E; (F) the original gels showing β-actinexpression in Fig 1E. Since our original exposure was very bright, there was no way to changeit, and we could not see the edges of the gels by adjusting the contrast in theoriginal gels of western blot. [file 12891_2022_5567_MOESM1_ESM.docx]

**Supplementary Figure 1. Isotype controls for immunofluorescence staining and immunohistochemistry staining and raw data of western blot in Figure 1.** (A) representative photo of the isotype control for immunofluorescence staining; (B) representative photo of the isotype control for immunohistochemistry staining; (C) the original gels showing TDO2 expression in Fig 1C; (D) the original gels showing β-actin expression in Fig 1C. (E) the original gels showing TDO2 expression in Fig 1E; (F) the original gels showing β-actin expression in Fig 1E.

Since our original exposure was very bright, there was no way to change it, and we could not see the edges of the gels by adjusting the contrast in the original gels of western blot.


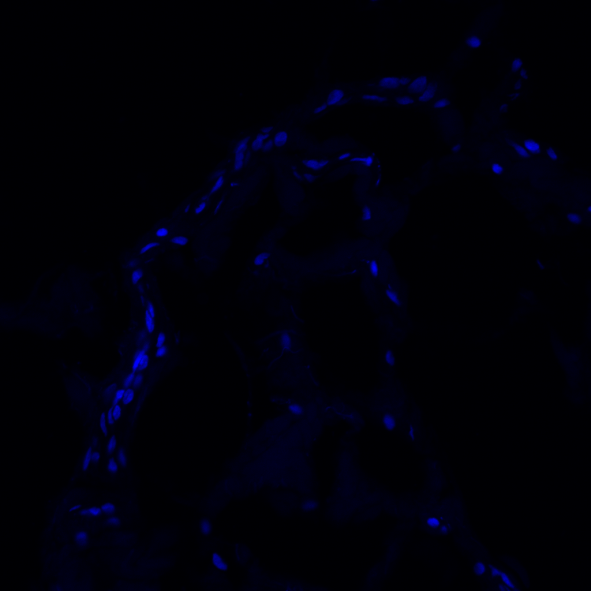


**Supplementary Figure 1A**. Representative photo of the isotype control for immunofluorescence staining in Fig 1A.


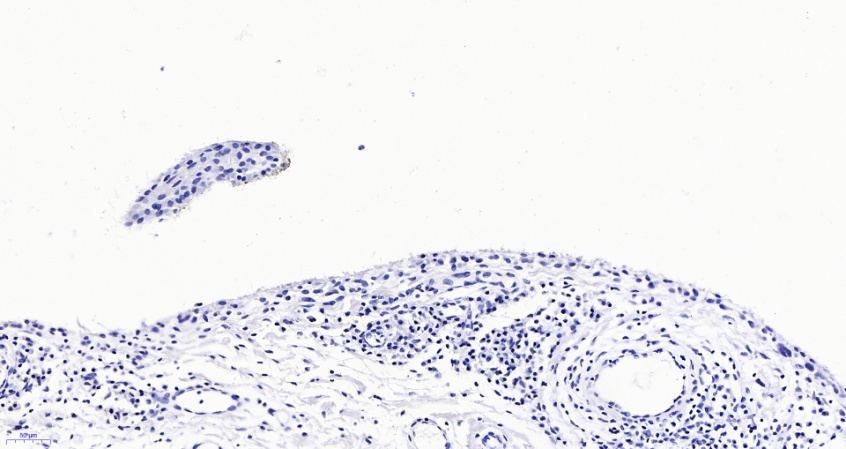


**Supplementary Figure 1B**. Representative photo of the isotype control for immunohistochemistry staining in Fig 1B.


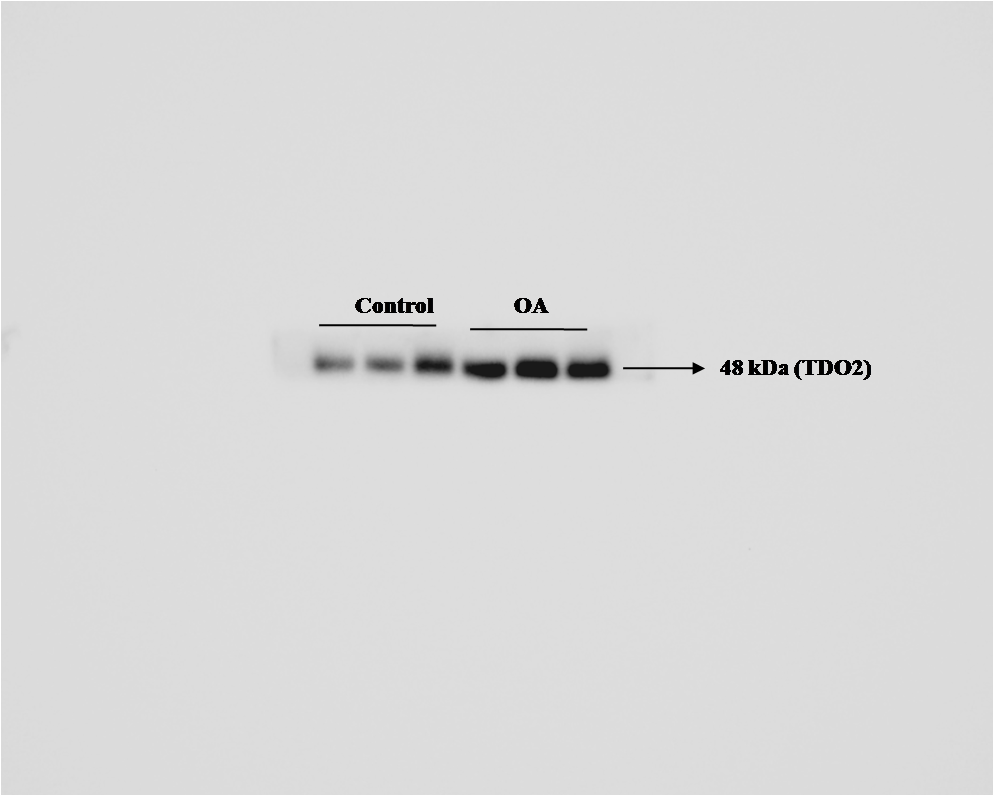


**Supplementary Figure 1C**. The original gels showing TDO2 expression in Fig 1C.


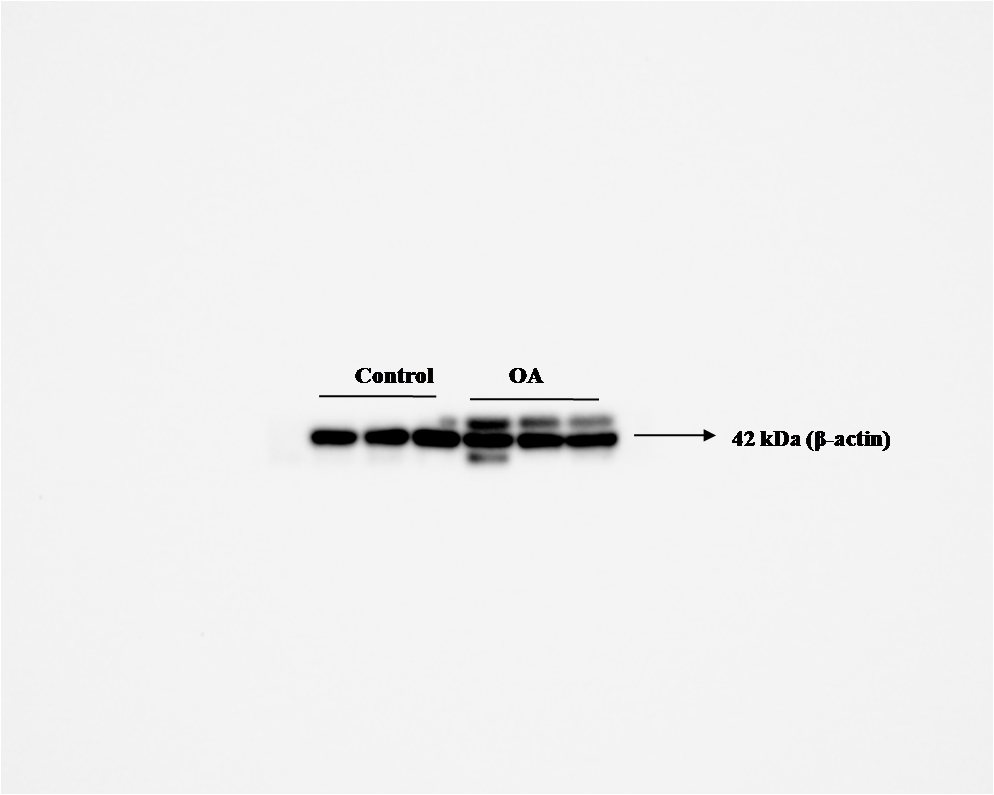


**Supplementary Figure 1D**. The original gels showing β-actin expression in Fig 1C.


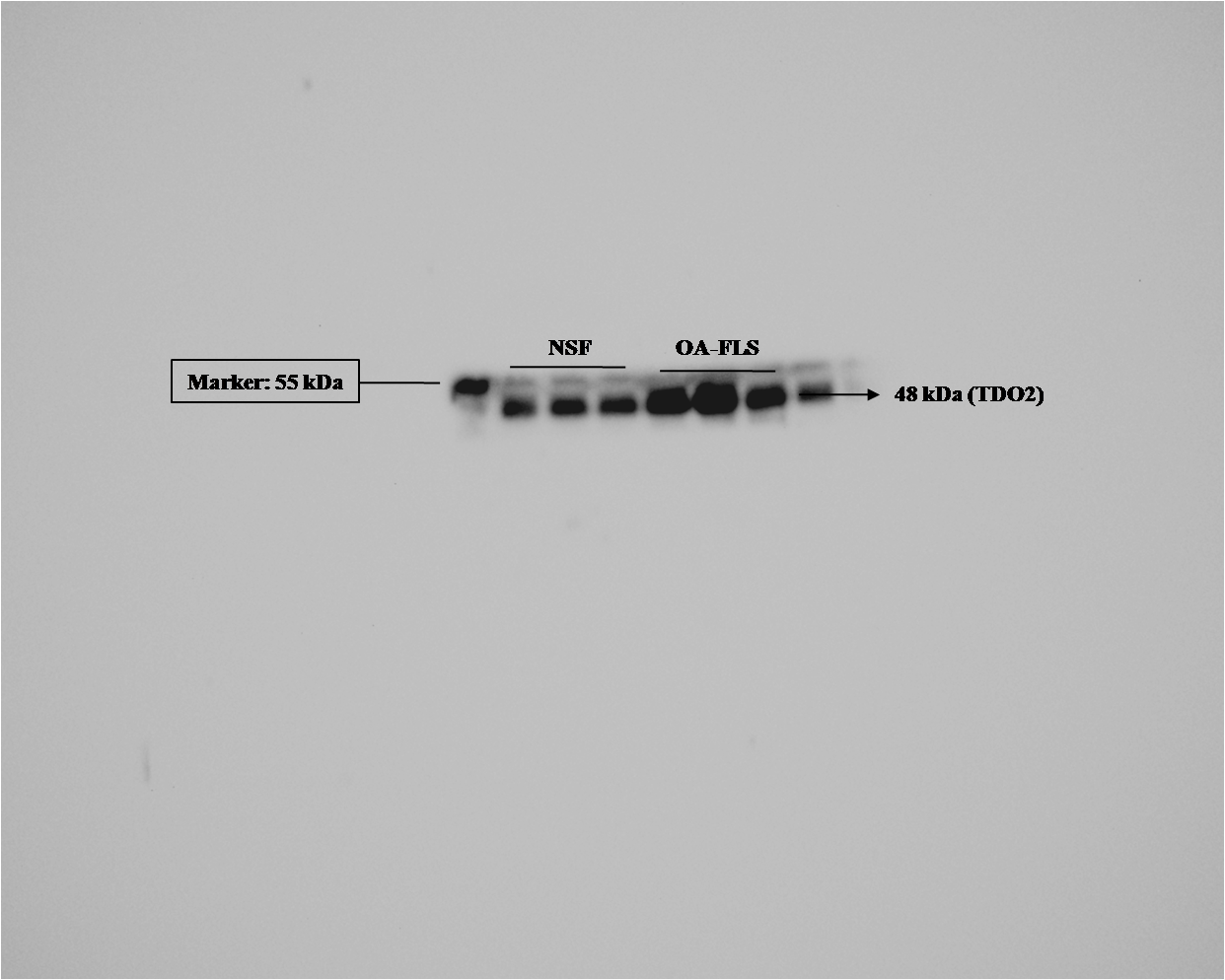


**Supplementary Figure 1E**. The original gels showing TDO2 expression in Fig 1E.


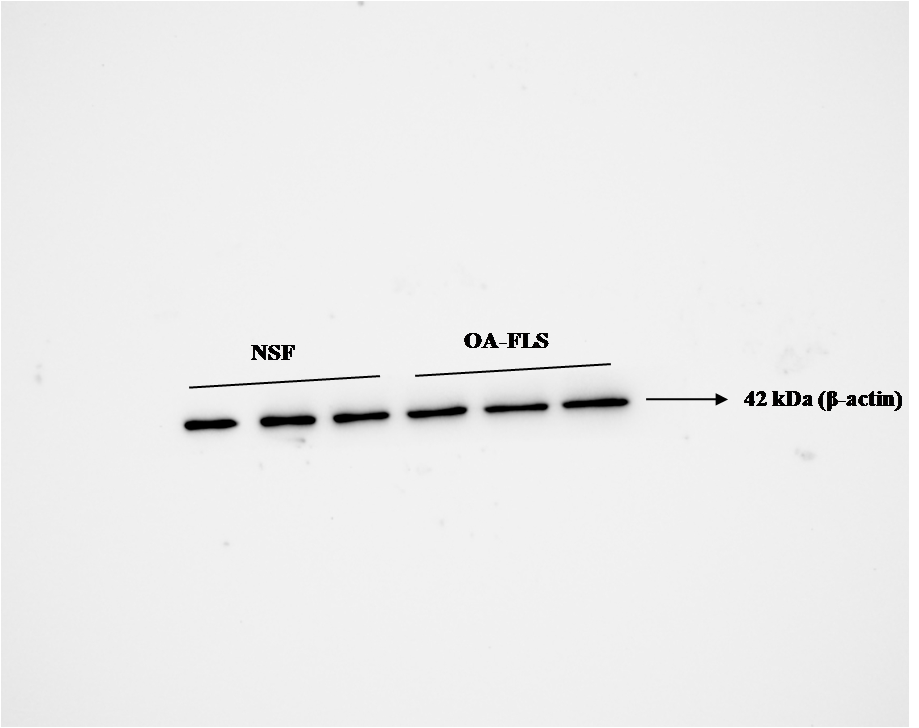


**Supplementary Figure 1F**. The original gels showing β-actin expression in Fig 1E.
