# Supplementary material for: High levels of TDO2 in relation to pro-inflammatory cytokines in synovium and synovial fluid of patients with osteoarthritis
Source: BMC Musculoskelet Disord. 2022 Jun 22;23:604. doi: 10.1186/s12891-022-05567-4 (PMC9214984; doi:10.1186/s12891-022-05567-4)
Supplement: Supplementary file 2 — Additional file 2: SupplementaryFigure 2. Raw data of western blot in Figure 4. (A) the original gels showing TDO2 expression inFig 4A; (B) the original gelsshowing β-actin expression in Fig 4A; (C) the original gels showing TDO2 expression in Fig 4B; (D) the original gels showing β-actinexpression in Fig 4B. [file 12891_2022_5567_MOESM2_ESM.docx]

**Supplementary Figure 2. Raw data of western blot in Figure 4.** (A) the original gels showing TDO2 expression in Fig 4A; (B) the original gels showing β-actin expression in Fig 4A; (C) the original gels showing TDO2 expression in Fig 4B; (D) the original gels showing β-actin expression in Fig 4B.


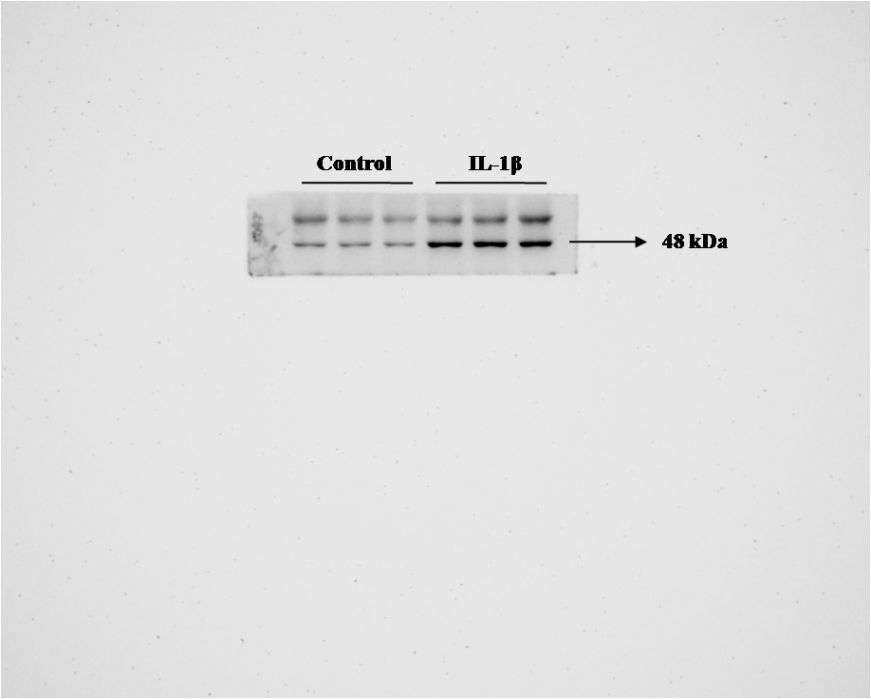

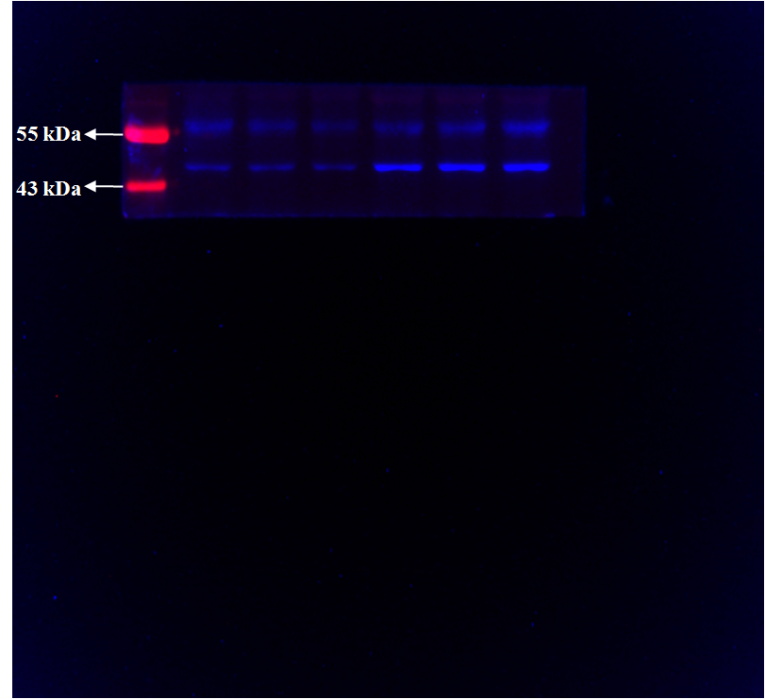


**Supplementary Figure 2A**. The original gels showing TDO2 expression in Fig 4A. (Thermo Scientific PageRuler Prestained Protein Ladder, #26635, marker was used)


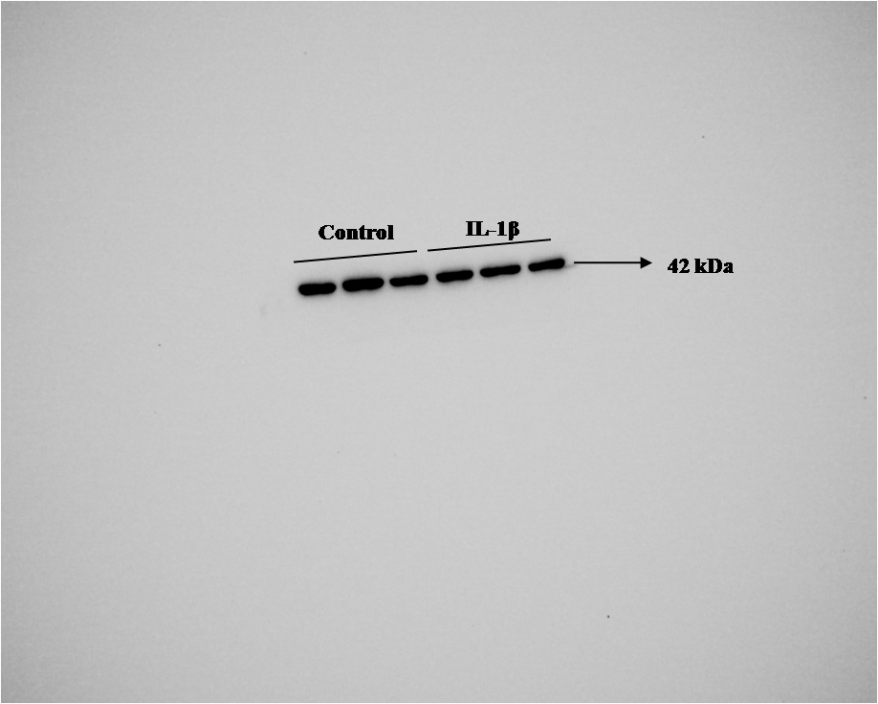

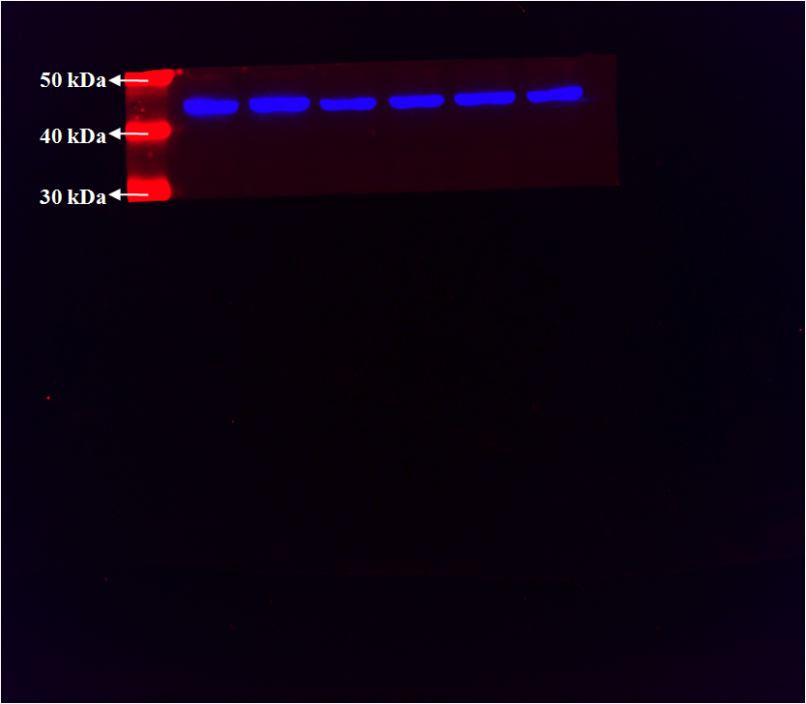


**Supplementary Figure 2B**. The original gels showing β-actin expression in Fig 4A.

(Thermo Scientific PageRuler Prestained Protein Ladder, #26630, marker was used)


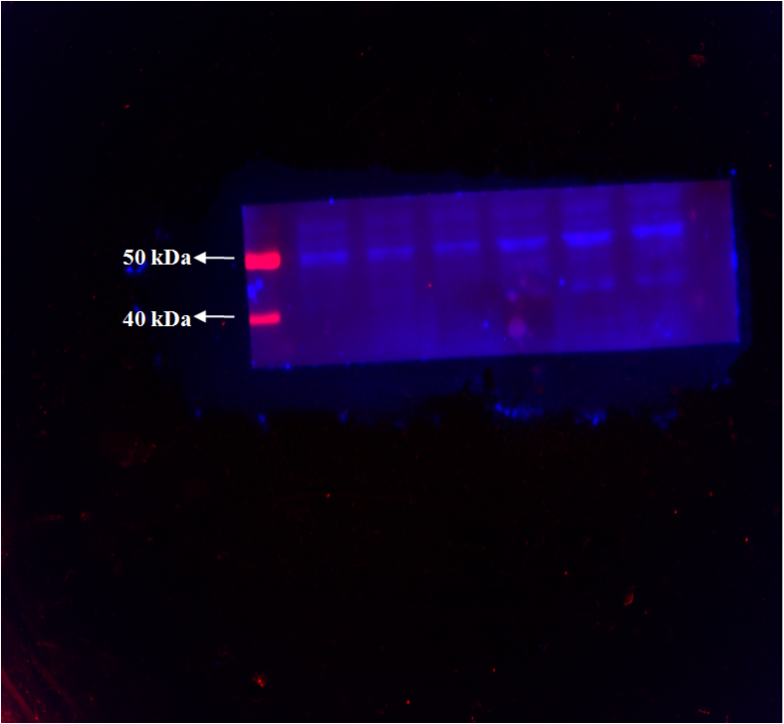

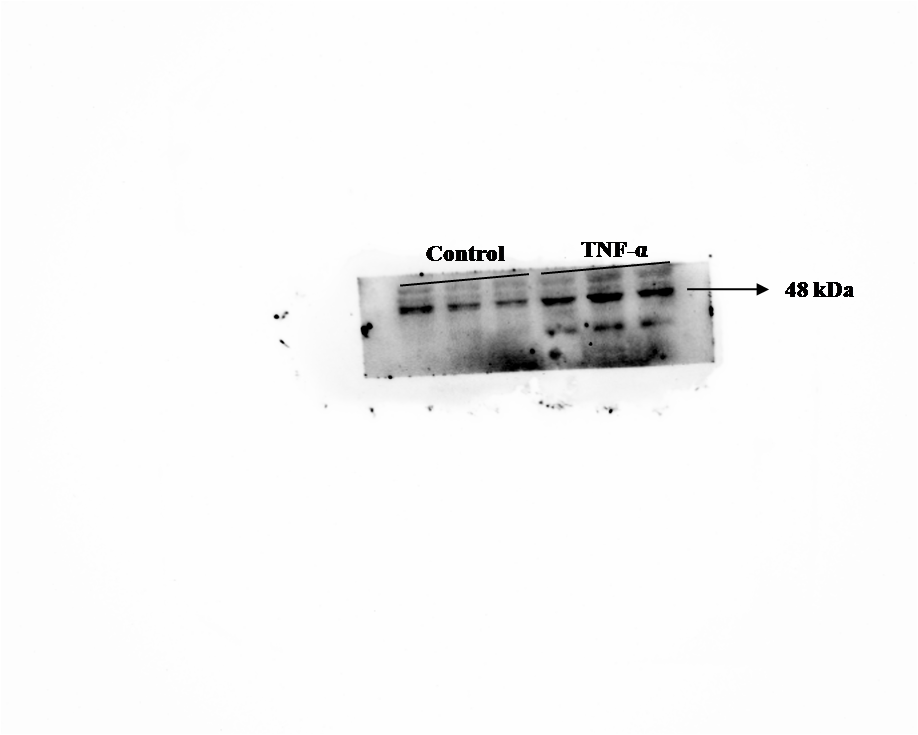


**Supplementary Figure 2C**. The original gels showing TDO2 expression in Fig 4B.

(Thermo Scientific PageRuler Prestained Protein Ladder, #26630, marker was used)


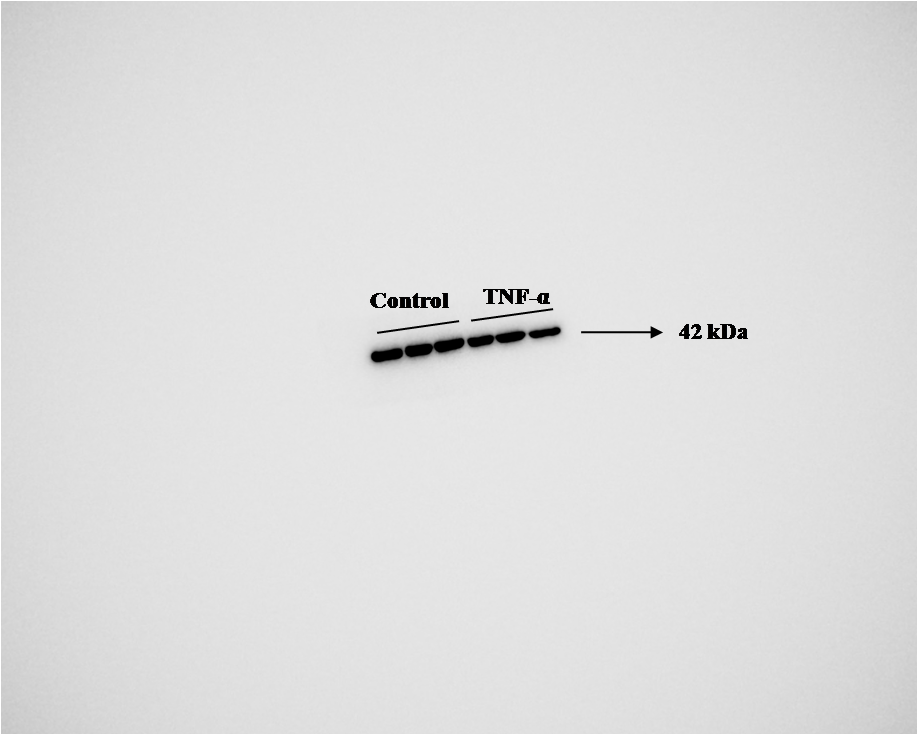


**Supplementary Figure 2D**. The original gels showing β-actin expression in Fig 4B.
